# Dietary Cows’ Milk Protein A1 Beta-Casein Increases the Incidence of T1D in NOD Mice

**DOI:** 10.3390/nu10091291

**Published:** 2018-09-12

**Authors:** Joanne S. J. Chia, Jennifer L. McRae, Ashwantha Kumar Enjapoori, Christophe M. Lefèvre, Sonja Kukuljan, Karen M. Dwyer

**Affiliations:** 1 Immunology Research Centre, St. Vincent’s Hospital, Fitzroy, Victoria 3065, Australia; joannesj.chia@gmail.com (J.S.J.C.); jennifer.mcrae@svha.org.au (J.L.M.); 2 Metabolic Research Unit, School of Medicine, Deakin University, Geelong, Victoria 3216, Australia; ashwantha.enjapoori@deakin.edu.au; 3 Division of Bioinformatics, The Walter and Eliza Hall Institute of Medical Research, Parkville, Victoria 3000, Australia; lefevre.c@wehi.edu.au; 4 Department of Pharmacology and Therapeutics, The University of Melbourne, Melbourne, Victoria 3010, Australia; 5 Peter MacCallum Cancer Centre, Melbourne, Victoria 3010, Australia; 6 Freedom Foods Group Ltd., Sydney, New South Wales 2229, Australia; skukuljan@ffgl.com.au; 7 Department of Nephrology, St. Vincent’s Health, Melbourne, Victoria 3065, Australia

**Keywords:** type 1 diabetes, beta-casein, cows’ milk, epigenetics, NOD mice

## Abstract

The contribution of cows’ milk containing beta-casein protein A1 variant to the development of type 1 diabetes (T1D) has been controversial for decades. Despite epidemiological data demonstrating a relationship between A1 beta-casein consumption and T1D incidence, direct evidence is limited. We demonstrate that early life exposure to A1 beta-casein through the diet can modify progression to diabetes in non-obese diabetic (NOD) mice, with the effect apparent in later generations. Adult NOD mice from the F0 generation and all subsequent generations (F1 to F4) were fed either A1 or A2 beta-casein supplemented diets. Diabetes incidence in F0–F2 generations was similar in both cohorts of mice. However, diabetes incidence doubled in the F3 generation NOD mice fed an A1 beta-casein supplemented diet. In F4 NOD mice, subclinical insulitis and altered glucose handling was evident as early as 10 weeks of age in A1 fed mice only. A significant decrease in the proportion of non-conventional regulatory T cell subset defined as CD4^+^CD25^−^FoxP3^+^ was evident in the F4 generation of A1 fed mice. This feeding intervention study demonstrates that dietary A1 beta-casein may affect glucose homeostasis and T1D progression, although this effect takes generations to manifest.

## 1. Introduction

Type 1 diabetes (T1D) results from the autoimmune destruction of insulin-producing beta cells in the pancreatic islets of Langerhans [1,2,3], culminating in the loss of blood-glucose homeostasis. T1D poses a serious health problem: the inability to regulate blood glucose levels necessitates exogenous insulin for survival; however, suboptimal glycemic control may lead to long-term complications resulting in substantial disability and reduced lifespan [4]. The International Diabetes Federation estimated that there were 437,500 children that have diabetes worldwide in 2007. Of all individuals with T1D, 70,000 young children under the age of 14 years, developing per year and the incidence will rise by 3% globally [5,6]. The data was significant as it comes from a large childhood T1D registry of 44 centres representing most countries in Europe [6]. T1D is a global disease, although there is geographical variation with respect to incidence and prevalence [7]. The cause of geographical variation could be due to differences in genetic and environmental risk factors [8]. 

Genetic predisposition, immunological and environmental factors such as dietary factors [9,10,11], infections [12], viruses [13] and gut microbiota [14,15] are all involved in the initiation, development and progression of T1D [5,7,16]. Studies of T1D genetics have revealed that individuals with specific human leukocyte antigen (HLA) genotypes, HLA DR and HLA DQ genotypes have an increased risk of developing the disease [17,18]. However, not everyone who has this genetic predisposition develops T1D, suggesting that environmental factors are needed to trigger and drive the disease [5,19]. Cows’ milk, one of the first foods introduced early to infants, is one such putative environmental factor [20]. Indeed, the identification of T1D-associated autoantibodies as biomarkers of pre-symptomatic disease in the birth cohort study, Diabetes Autoimmunity Study in the Young (DAISY), found that children who have the low to moderate HLA-DR genotype paired with a greater dietary intake of cows’ milk protein may be at an increased risk of developing islet autoimmunity and progression to T1D [21]. More recently, results of a randomized trial to reduce insulin dependent diabetes in the genetically at risk (TRIGR type 1 diabetes primary prevention pilot study) reported that cows’ milk consumption was associated with increased risk of beta-cell autoimmunity and T1D in children with genetic susceptibility [22].

The World Health Organization (WHO) recommends that infants be exclusively breast fed for six months and breastfeeding should continue beyond the second year to ensure healthy growth and development [23,24]. Beyond weaning, cows’ milk is introduced into the diets of infants [25]. Cows’ milk itself is introduced into the diet of infants as they age. Beta-casein is one of the major proteins contained in cows’ milk and constitutes up to 35% of the total protein in cows’ milk [26]. Currently, thirteen beta-casein genetic variants have been identified [27]. The most common are the A1 and A2 genetic variants, the former differing from the latter in one amino acid substitution (Pro_67_ to His_67_) [28]. The amino acid substitution is associated with physiochemical properties of A1 beta-casein digestion at position 67. During *in-vivo* and *in vitro* digestion, only the A1 variant produces a seven amino acid peptide called beta-casomorphin 7 (BCM-7) [29,30,31]. The impact of BCM-7 on human disease, in particular T1D, is the subject of intense debate [32,33,34,35].

Most compelling is the data analysis by Laugesen and Elliott, which demonstrated a positive correlation (*r* = 0.92) between cows’ milk A1 beta-casein supply per-capita and T1D in 19 developed countries [36]. The 19 countries included in the analysis were the USA, Canada, Venezuela, Oceania (Australia and New Zealand), East Asia (Japan) and Middle East (Israel). A higher incidence rate was observed in Finland and Sweden (highest A1 β-casein consumption/per capita) and very low rates have been found in Venezuela and Japan (lowest A1 β-casein consumption/per capita) [36].

The association between beta-casein consumption and T1D has been investigated in rodent models although mechanisms have been difficult to define. Two publications highlighted the relationship between cows’ milk consumption and T1D. Firstly, in 1997, Elliot et al. reported that NOD mice fed a 2% casein supplemented diet at weaning developed T1D at a greater rate than NOD mice fed base (Pregestimil powder) diet (14.6% versus 1% at 250 days) [37]. Later, in 1997, Elliot et al. reported that a 28% of female NOD mice fed whole A1 beta-casein developed T1D at 250 days compared with 2% on the Pregestimil diet [38]. Given the controversy surrounding the purported association between A1 beta-casein consumption and T1D, we sought to test whether a diet supplemented with A1 or A2 beta-casein would increase the incidence of T1D in genetically susceptible female NOD mice over generations.

## 2. Materials and Methods

### 2.1. Animal Experiments

Newly weaned 3–4 week old male and female NOD/ShiLtJArc mice were obtained from the Animal Recourses Centre, Canning Vale, Western Australia, Australia. Mice were housed in a pathogen-free environment in the Experimental Medical Surgery Unit, St Vincent’s Hospital, Melbourne. These mice (designated F0) were immediately separated into two cohorts and fed a nutritionally balanced milk-based diet containing either the A1 or A2 beta-casein component. The diets were prepared by Specialty Feeds (Glen Forrest, Western Australia, 6071) (Table 1), in accordance with strict manufacturing protocols. Feeds were produced every three months and stored under strict temperature controlled environments, in order to ensure that the quality and freshness of the diets was maintained.

Mice were fed ad libitum and had free access to drinking water. Non-fasting blood glucose levels (BGLs) were monitored weekly throughout the 30-week study. 

All animal experiments in this study were approved by the St. Vincent’s Hospital Animal Ethics Committee (Melbourne, Australia).

### 2.2. Breeding Program

For breeding further generations, 6–8 week old mice were mated. Brother/sister breeding pairs from the A1- and A2-fed F0 cohort were mated to generate F1 offspring. Similarly, breeding pairs from the F1 generation were mated to produce the F2 generation. Two further generations (i.e., F3 and F4) were produced. This resulted in all offspring from F1 to F4 being exposed to either A1 or A2 beta-casein only from conception.

### 2.3. Blood Glucose Monitoring and Diabetes Incidence

Weekly BGLs were monitored from 6 to 30 weeks of age. Mice were deemed diabetic if they had a reading of more than 20 mM. Diabetes incidence at any week in time was determined by the formula:

(1) $$Diabetes\;incidence=\frac{Number\;of\;mice\;fed\;a\;particular\;beta-casein\;diet\;with\;BGL\;>\;20\;\mathrm{mM}}{Total\;number\;of\;mice\;fed\;a\;particular\;beta-casein\;diet}.$$

### 2.4. Intraperitoneal Glucose Tolerance Test

Female NOD mice at 10- to 12-weeks old from the F4 generation were made to fast overnight (12–16 h). The mice were injected intraperitoneally with glucose (2 g/kg body weight). BGLs were measured from blood samples collected from the tail at 0, 15, 30, 45, 60, 90, and 120 min.

### 2.5. Insulin Tolerance Test

Insulin (0.75 IU/kg body weight) was administered intraperitoneally into 10-week old female mice from the F4 generation after a 12-h overnight fast. BGLs were determined from blood samples obtained from the tail at 0, 15, 30, 45, 60, 90, and 120 min after the injection. 

### 2.6. Immune Profiling

Leukocytes were obtained from peripheral lymphoid organs (spleen, thymus, pancreatic and mesenteric lymph nodes) and blood. Red blood cells were cleared using 0.9% ammonium chloride solution. The leukocytes were then washed and spun down and were stained with antibodies for various leukocyte populations. The antibodies used were as follows: Fc block (anti-mouse FcRγIII/II mAb, 2.4G2, BD Biosciences, Franklin Lakes, NJ, USA), CD3-FITC (Clone: 17A2, BD Biosciences), CD4-PeCy5 (Clone: H129.19, BD Biosciences), CD8-APC-Cy7 (Clone: 53-6.7, BD Biosciences), CD19-PeCy7 (Clone: 1D3, BD Biosciences), CD25-APC-Cy7 (Clone: PC61, BD Biosciences), CD45 Pacific Blue (Clone: 30-F11, Biolegend, San Diego, CA, USA), and F4/80-APC (Clone: BM8, Invitrogen, Carlsbad, CA, USA). For intracellular FoxP3 staining, cells were incubated with FcRγIII/II, surface stained for CD4 and CD25, fixed and permeabilised as per manufacturer’s instructions (eBioscience, Waltham, MA, USA), and stained for FoxP3 (Clone: FJK-16S, eBioscience). Cells were then analysed using a FACSCanto flow cytometer (BD Biosciences) and Diva software (BD Biosciences). A total of 1 × 10^6^ cells were analysed. 

### 2.7. Treg Suppression Assays

Mouse CD4^+^CD25^+^ Treg and CD4^+^CD25^−^ Tresp populations were purified using the CD4^+^CD25^+^ Regulatory T cell isolation kit (Catalog number: 130-091-041) and AUTOMACS (Miltenyi Biotec Australia, Macquarie Park, NSW, Australia) as per manufacturer’s instructions. Tresp were stained with CFSE using the CellTrace™ CFSE Cell Proliferation Kit (Molecular Probes™, Thermo Scientific, Melbourne, VIC, Australia). Accessory cells were irradiated CD4-depleted splenocytes. In each well, a total of 1 × 10^5^ Tresp cells and 1 × 10^5^ accessory cells were incubated with 1 μg/mL anti-CD3 antibody (WEHI) in complete medium (RPMI-1640 containing Glutamax, 1 mM Pen-Strep, 1 mM Na-Pyruvate (Gibco™, Thermo Scientific, Melbourne, VIC, Australia), 10% mouse sera and 50 μM 2-mercaptoethanol (Sigma-Aldrich, Castle Hill, NSW, Australia)). To assess suppressive activity, Treg were co-cultured at Treg: Tresp ratios of 1:1 to 1:16 for 3 days. Following culture, wells were washed and cells were incubated with anti-mouse CD4-APC Ab (Clone: RM4-5, eBiosciences) at room temperature. 7AAD (BD Biosciences) was added to exclude dead cells prior to FACS analysis. CD4^+^ cells were gated and Tresp proliferation assessed using FlowJo v7.6 Proliferation Platform (Tree Star, Ashland, OR, USA), on a FACSCanto (BD Biosciences). 

### 2.8. Peptide Extraction from Whole Blood, Lymph Tissues and Mass Spectrometry

Whole blood samples from female NOD mice fed on A1 diet (each generation F0–F4; *n* = 10) were collected in vials containing dipeptidyl peptidase -IV inhibitor, immediately aliquoted and stored at −80 °C until the time of analysis. The peptides were extracted from whole blood using a previously described procedure [39]. Peptide analysis was carried out on a QExactive plus Orbitrap mass spectrometer (Thermo Scientific).

Female NOD mice from F0 generation (*n* = 12) mesenteric and pancreatic lymph nodes were collected in vials and immediately stored at −80 °C until the time of analysis. In brief, the tissues samples were finely diced using a scalpel and then homogenised on ice in 600 µL of ice-cold lysis buffer (10 mM Tris, pH 7.5; 25 mM KCl; 250 mM sucrose; 1 mM EDTA; 150 mM NaCl; 1 mM PMSF). The samples were left for 30 min and then spun at 12,000× *g* for 15 min at 4 °C to pellet any insoluble material. For peptide recovery, 500 µL of tissue supernatant were mixed with 1 mL of acidic acetonitrile and acetone, containing individual labelled internal standard for each targeted beta-casomorphin peptide (BCM-5, BCM-7, and BCM-9). The mixtures were spun at 12,000× *g* for 15 min at 4 °C and 1200 µL of supernatant from each extract was recovered to a new Lo-bind tube, evaporated to dryness, and reconstituted for HLB Prime solid phase extraction (SPE) clean-up. Peptides recovered from the SPE clean-up were again evaporated to dryness and reconstituted for the final analysis by LC-MS/MS (Sciex 6500 TripleQuad). LC separation was performed on a Water XSelect^®^ HSS T3 2.5 µm column (2.1 × 50 mm). The mass spectrometer was operated in SRM mode, alternating between detection of BCM-5, BCM-7, and BCM-9 transition ions.

### 2.9. Bacterial DNA Preparation

Faecal samples were collected from F0 generation female NOD mice (A1 diet *n* = 6; A2 diet *n* = 6) and stored at −80 °C immediately after collection until further use. Genomic DNA from faeces was extracted using a QIAamp DNA stool mini kit (Qiagen, Germantown, MD, USA) according to the manufacturer’s instructions, with some modifications. Briefly, an aliquot (~100 mg) of each fecal sample was suspended in 1 mL of inhibit EX buffer. Microbial cells were then lysed by mechanical disruption with a TissueLyser II (Qiagen) for 3 min at 30 Hz. After centrifugation, the supernatant was digested with proteinase K and DNA was precipitated with ethanol. DNA was further washed and purified by a QIAamp spin column. DNA was eluted in 0.2 mL of elution buffer ATE. The quality and quantity of the genomic DNA was measured using the NanoDrop assay. DNA concentrations were adjusted to 100 ng/μL for subsequent Metagenome shotgun pyrosequencing. 

### 2.10. High-Throughput Sequence Analysis

Sequencing was performed by Macrogen (Seoul, Korea) using paired-end sequencing with read length 101 nucleotides using TruSeq Nano DNA kit sample library preparation protocol (Part #15041110 Rev. A) on a HiSeq 25000 System (Part #15011190 Rev. V HCS 2.2.70). For each of the six samples from mice fed the A1 and A2 diets, 8 to 12 million reads were obtained. The data was annotated using the Centrifuge software and a mapping threshold of 80 nucleotides minimum match length was apply to filter poor confidence matches [40]. Results were analysed with the R packages Phyloseq [41] and DESeq2 version 1.2.10 [42,43].

### 2.11. Histological Staining

Intestinal sections from the jejunum, proximal ileum and distal ileum and pancreatic tissues of F4 generation NOD mice were collected. These tissues were fixed in 10% phosphate-buffered formalin overnight at room temperature and embedded in paraffin. Sections (intestinal: 1–2 μm, pancreatic: 4 μm) were stained with hematoxylin and eosin (H&E; Merck, Darmstadt, Germany) using standard techniques. H&E-stained tissue sections were visualized and evaluated under a standard light microscopy using an AxioImager Z1 microscope (Carl Zeiss MicroI-maging, Jena, Germany). 

### 2.12. Statistical Analysis

Results are reported as mean ± standard error of the mean (SEM). Statistical analysis was performed by one-way ANOVA and Student’s *t*-test. *p* < 0.05 was considered a statistical significance between groups.

## 3. Results

### 3.1. Effect of A1 Beta-Casein Supplemented Diet on Incidence of T1D in NOD Mice

**Figure 1:**
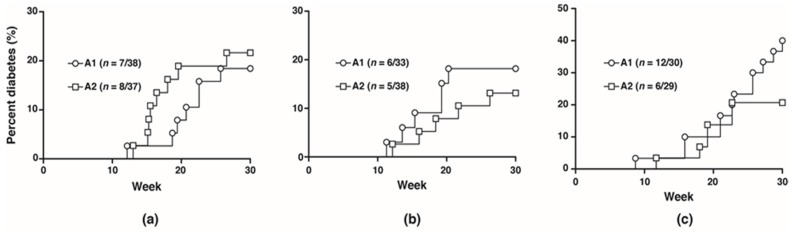
Diabetes incidence in female mice. Diabetes incidence of mice in the (**a**) F1 generation; (**b**) F2 generation; (**c**) F3 generation.

To investigate the potential effects of the A1 and A2 beta-casein on the development of T1D, NOD mice were fed with either diet separately for five generations and monitored for 30 weeks. No difference in diabetes incidence was observed between the two cohorts from F0 to F2 generations (F1: A1 18.4% vs. A2 21.6%; F2: A1 18.2% vs. A2 13.2%). In the F3 generation, at 30 weeks, the diabetes incidence was doubled in the cohort fed A1 beta-casein compared to the A2 cohort (A1: 40% vs. A2: 20.7%) (Figure 1). 

**Figure 2:**
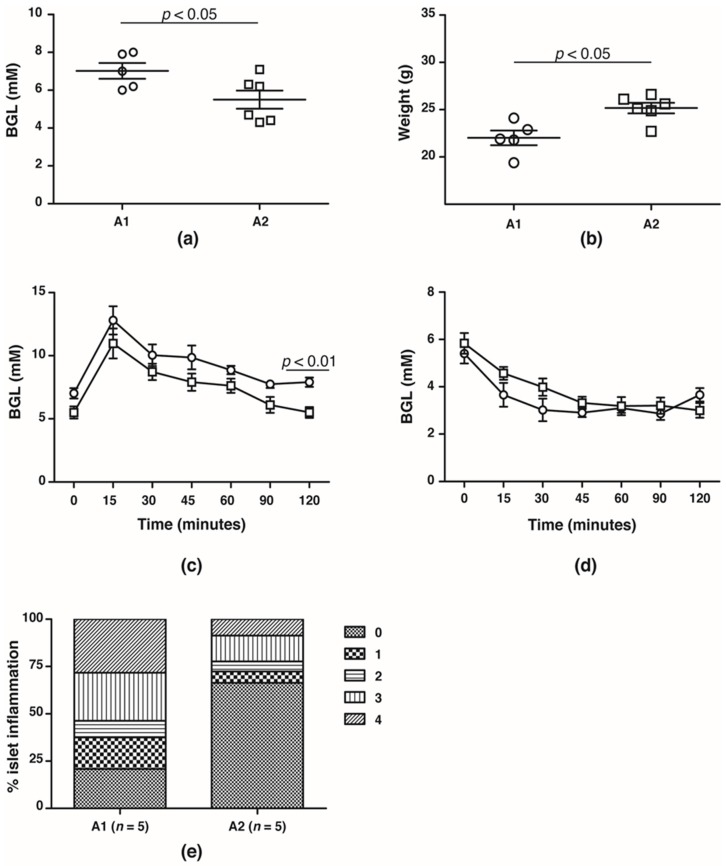
Glucose handling capacity in F4 generation female NOD mice. (**a**) fasting  blood glucose levels and (**b**) body weights of 10- to 12-week old A1 (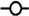) and A2 (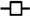) beta-casein fed female mice. BGLs of female mice assessed in (**c**) glucose and (**d**) insulin tolerance tests. (**e**) distribution of insulitis in islets represented as a percentage of islet infiltration in 10-week old female mice fed either A1 or A2 beta-casein supplemented diets. Islets were scored blindly from individual mice. Islet inflammation was calculated on a scale from 0 to 4. 0—islets devoid of mononuclear cells; 1—minimal (<10%) focal islet infiltrate; 2—peri-islet infiltrate in <25% of islet circumference; 3—peri-islet infiltrate in >25% but <50% intra-islet and 4—>50% intra-islet infiltration.

In the F4 generation, the glucose handling capacity was assessed prior to the onset of T1D at 10 weeks of age. Fasting BGLs was significantly higher in NOD mice fed the A1 beta-casein supplemented diet compared with mice fed the A2 beta-casein supplemented diet (A1: 7.0 ± 0.4 mM vs. A2: 5.5 ± 0.5 mM, *p* < 0.05) (Figure 2a). This was associated with lower body weights in the cohort fed an A1 beta-casein supplemented diet (A1: 21.5 ± 0.6 g vs. A2: 23.6 ± 0.3 g, *p* < 0.05) (Figure 2b). NOD mice fed A1 beta-casein had higher 2-h BGLs compared to A2 beta-casein fed mice (A1: 7.9 ± 0.4 mM vs. A2: 5.5 ± 0.4 mM, *p* < 0.05) (Figure 2c). In both cohorts, glycemic response to insulin tolerance testing was preserved (Figure 2d) indicating normal insulin sensitivity. Insulitis was evident in 80% of islets graded in the A1 beta-casein fed mice and among those, 55% were graded as severe with a grade 3 or 4, whereas the majority of islets (~70%) from the A2 beta-casein cohort were free from insulitis (Figure 2e). Together, these data suggest that an A1 beta-casein diet alters glucose handling capacity by promoting islet inflammation.

**Figure 3:**
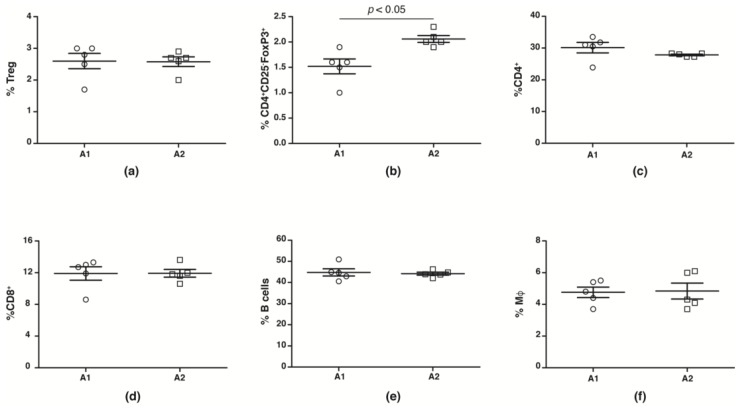
Immune profile in F4 generation mice fed A1 and A2 beta-casein supplemented diets. Splenic leukocytes were obtained and stained with various antibodies. The percentages of the different immune subsets in the spleen were then assessed via flow cytometry. (**a**) conventional Tregs (CD4^+^CD25^+^FoxP3^+^); (**b**) non-conventional Tregs (CD4^+^CD25^−^Foxp3^+^); (**c**) CD4^+^; (**d**) CD8^+^; (**e**) B cells; and (**f**) macrophages.

While human studies report no change in peripheral blood Treg numbers, suppressive capacities are reduced [44,45,46,47,48]. We evaluated splenic CD4^+^CD25^+^FoxP3^+^Treg, CD4^+^CD25^−^FoxP3^+^ Treg, macrophage, CD4^+^, CD8^+^ and B cell numbers from NOD mice fed with A1 and A2 beta-casein supplemented diet across the generations. We observed no change in number (Figure 3a) and function (Supplementary Figure S1) of conventional CD4^+^CD25^+^FoxP3^+^Tregs in the F4 generation NOD mice fed with A1 and A2 diets. However, there was a significant decrease in the Treg subset defined by CD4^+^CD25^−^FoxP3^+^ in the A1-fed mice compared to the A2-fed cohort (Figure 3b). The numbers of CD4^+^, CD8^+^, B cells and macrophages were unaltered (Figure 3c–f).

### 3.2. Isolation and Analysis of Peptides from Whole Blood and Lymph Tissues by Mass Spectrometry

To test for the presence of BCM-7 peptide in the whole blood and lymph tissues of NOD mice, peptides were isolated and analysed by LC-MS/MS and triple-quadrupole mass spectrometry, respectively. No BCM-7 peptide was detected in either sample type (data not shown).

### 3.3. The Effect of A1 Beta-Casein Supplemented Diet on Intestinal Microbial Communities of Female NOD Mice in the F0 Generation

To test whether A1 beta-casein supplemented diet altered the gut microbiota in mice, we performed metagenome shotgun sequencing on the faecal samples obtained from the F0 generation of female NOD mice fed A1 and A2 supplemented diets for six weeks. There were small differences in bacterial diversity (Supplementary Figure S2), but no significant differences in microbial abundances at the phylum level between the A1 and A2 diet groups. We did find some operational taxonomic units (OTUs) that exhibited a trend towards differential expression. Compared to A2 beta-casein fed NOD mice, A1 beta-casein supplementation increased the level of several bacterial species, some of which have been shown to produce effects on diabetes, including *Streptococcus pyogenes* [49] and *Streptococcus suis*. [50]. In contrast, *Enterobacter cloacae*, *Enterobacter hormaechei* and *Klebsiella oxytoca* at the species level were reduced by A1 beta-casein supplemented diet. 

### 3.4. A1 Beta-Casein Diet Consumption Did Not Alter the Gastrointestinal Integrity in Female NOD Mice in the F4 Generation

In mice, a leaky gut with increased gut permeability, disturbed microbial balance and impaired intestinal mucosal immunity are associated with T1D [51]. The impact of A1 and A2 supplemented diets on the cellularity and architecture of the small intestine in the NOD F4 mice was assessed histologically. Neither an A1 nor A2 supplemented diet impacted the gastrointestinal integrity of the mice (data not shown).

## 4. Discussion

Despite a number of reports demonstrating a positive correlation between dairy consumption and risk of T1D [36,52,53], conclusive evidence of causation is lacking. This feeding intervention study demonstrates that the consumption of A1 beta-casein in genetically susceptible mice increases the incidence of T1D, which only becomes evident in later generations. We chose to study female NOD mice as these mice have a heightened susceptibility to T1D [54,55,56] and monitored mice for a number of generations. Later generations showed an increased incidence of T1D, altered glucose handling and associated weight loss and a reduction in a non-conventional Treg cell subset.

The main finding in this study was the doubling of T1D incidence in the F3 generation and presence of subclinical insulitis in 10-week old F4 female NOD mice, suggesting the onset of T1D is influenced by an epigenetic phenomenon [57,58]. It has been reported that diet alone [59] and diet and microbiota mediated epigenetic programming can affect the expression of insulin resistance and insulin signalling genes [60]. In addition to genetic and environmental factors, epigenetic modifications may contribute to the etio-pathogenesis of T1D, necessitating studies with long follow-up durations to capture incidence and enable identification of dietary associations. Indeed, inconsistent findings from some animal studies of A1 beta-casein diet supplementation and T1D associations might be a consequence of the very long latency for T1D development.

We found a reduction in the CD4^+^CD25^−^Foxp3^+^ T cell subset. Although not currently described in the T1D setting, changes in this population have been described in autoimmune systemic lupus erythematosus during active disease [61,62]. Questions as to their role remain, however these ‘atypical’ Tregs share similar expression profiles with CD4^+^CD25^+^FoxP3^+^ Tregs and display some suppressive capacity [61]. Plasticity in Treg populations subsets has been well described [63] and may contribute to the differences in proportions of CD4^+^CD25^+^FoxP3^+^ and CD4^+^CD25^−^FoxP3^+^ in this study. One study described CD4^+^CD25^−^FoxP3^+^ as a “replenishment pool” that can be recruited and regain their CD25 expression to help combat autoreactive immune cells following disease onset [64]. Thus, the CD4^+^CD25^−^FoxP3^+^ increase observed in the A2-fed NOD mice may represent an effort to regulate autoreactive immune cells. Further investigations are warranted to elucidate the nature of this T cell subset and their role in T1D.

Following A1 beta-casein milk or supplemented diet intake, the BCM-7 peptide has been detected in animal and human biofluids [31,65,66,67]. BCM-7 was not detected in peripheral blood and mesenteric lymph nodes of NOD mice fed an A1 supplemented diet by mass spectrometry. This may be due to lower than limit-of-detection levels of BCM-7 in the samples, timing of lymph node retrieval or the complex blood peptide samples. We are currently investigating whether BCM-7 has a direct effect on islet development.

Although others have shown changes in gastrointestinal architecture and inflammation after short periods of exposure to A1 beta-casein [68], we did not see such changes, even in the fifth generation of mice. The different rodent strains may account for these disparate findings—where the other studies were performed with male Swiss albino mice [69] and Wistar rats [70], our study utilised NOD mice.

Recently, it was reported that casein supplemented diets modulate the composition of rat gut bacteria [71,72]. The rats fed with casein had a higher abundance of Bacteroidetes at phylum level [72]. At the family level, the composition of bacteria had lowest abundance of *Lactobacillaceae* and highest of *Lachnospiraceae* [72]. The *Lactobacillaceae* members have been proposed to play a key role in host metabolic homeostasis by protecting the gut integrity against pathogens disruption and can reduce inflammation [72]. Intestinal inflammation is a recognised trigger of T1D [51]. We did not observe a significant change in gastrointestinal microbiome composition in F0 generation of mice, and due to funding limitations we did not collect faecal samples in the generations in which increased diabetes incidence was observed. Faecal samples from the later generations in which diabetes incidence was increased would provide greater insight into the potential role of the microbiome in the onset of T1D.

A positive association between food allergy and *Helicobacter pylori* infection has been reported [73], and children with *H. pylori* infection have been shown to exhibit elevated IgE responses to cow’s milk [74]. It is therefore possible that *H. pylori* infection at least partly explains the observed relationship between A1 beta-casein consumption and the increased incidence of T1D [36]. *H. pylori* infection reportedly increases epithelial permeability and permits the non-selective passage of allergens to cross the intestinal barrier [73]; a similar cascade of events could permit the passage A1 beta-casein derived BCM-7 to cross the epithelial barrier. If so, the previously reported immunological cross reactivity or molecular mimicry between an epitope on the pancreatic beta-cell-specific glucose-transporter GLUT-2 and the BCM-7 peptide may explain some of the interplay between T1D and A1 beta-casein [75]. Here, exposure to A1 beta-casein may promote the development of autoantibodies that ultimately contribute to the cascade of events leading to type 1 diabetes development. Autoantibodies to GLUT-2 have been detected in patients with recent onset T1D [76] and reactivity of beta-casein T-cell lines to human insulinoma extracts and GLUT-2 have been reported [77]. However, the full implications of these findings are open to speculation because beta-cell autoantibodies may not necessarily be pathogenic: rather, they may represent reproducible biomarkers of the pathogenesis.

This study had a number of limitations that should be considered. The cumulative incidence of diabetes in our NOD colony was low, impacted by in-house microbiota colonisation [54]. We only investigated changes in the microbiome from the F0 generation between A1 and A2 fed mice and data on BCM-7 detection is limited to lymph nodes and blood. The major strength of this study is that experimental conditions were stringently controlled throughout and diabetes incidence across multiple generations were analysed, which to our knowledge has not previously been done. 

**Figure 4:**
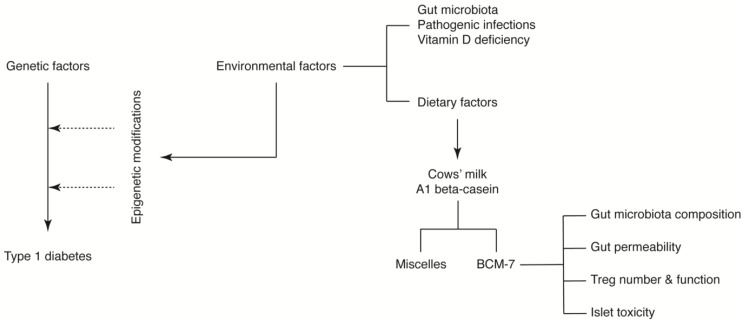
A1 beta-casein supplemented diet associated mechanisms in T1D development in NOD mice.

Despite the limitations described above, we propose that A1 beta-casein influences T1D incidence through a number of potential mechanisms mediated via BCM-7. We hypothesise that BCM-7 released from A1 beta-casein may influence the immune response [38], gut architecture and microbiota [71,72,78] and/or impart direct islet toxicity. Together, these effects may induce epigenetic alterations predisposing pancreatic beta-cells to an autoimmune response (Figure 4) [60,79].

The specific contribution of these mechanisms to the development of T1D, as well as their ability to compensate for each other, is unknown. Furthermore, in milk, caseins form complex aggregates with calcium phosphate called casein micelles [80]. Casein micelles are the source of calcium phosphate and proteins to the young for the growth of bone and teeth [81]. A recent study revealed that cows’ milk A1 beta-casein forms a larger micelle compared to A2 beta-casein, which may influence their function [82]. Whether casein micelles were operational in this model was not investigated.

## 5. Conclusions

The data presented in this study are provocative and suggest an interaction between dietary protein consumption and the incidence of T1D that differs across generations in NOD mice. This study, alongside others [36,37,38,83,84,85,86], raises questions regarding widespread consumption and timing of introduction of A1 beta-casein in early childhood. Many other environmental factors, such as infections, air pollution, vaccines, location of residence, family environment and stress [87], have been postulated as potential environmental triggers [11]. It is feasible that a combination of environmental triggers in the genetically susceptible host is required. Because cows’ milk is the only alternative source of nutrition after breast milk for neonatal feeding in the general population, definitive testing of the hypothesis is clearly warranted. 

## Figures and Tables

**Table 1:** The nutrient composition of the experimental A1 and A2 beta-casein supplemented diets for mice.

Ingredients (g/100 g)	A1A1 Skim Milk Diet	A2A2 Skim Milk Diet
Sucrose	25.753	25.753
Skim milk powder (as supplement)	60.528	60.529
Instruction	Standard mixing	
COPHA hydrogenated vegetable oil	1.366	1.366
Palm oil	3.709	3.709
Safflower oil (High Linoleic)	0.787	0.787
Flax oil	0.462	0.462
Instructions	Standard mixing	
Cellulose	5.000	5.000
Instructions	Standard mixing	
dl Methionine	0.904	0.904
A1N_93_Trace minerals	0.140	0.140
Salt (Fine sodium chloride)	0.100	0.100
AIN_93_Vitamins	1.000	1.000
Choline chloride 75% *w*/*w*	0.250	0.250
Red food colour	0.001	-
Instructions	Standard mixing	
Total	100.000	100.000

## Supplementary Materials

The following are available online at http://www.mdpi.com/2072-6643/10/9/1291/s1, Figure S1: Treg-mediated suppression in the F4 generation NOD mice, and Figure S2: The diversity of gut bacteria in faecal contents level of A1 and A2 beta-casein fed F0 NOD mice according to several diversity indices.
