# Supplementary material for: Dietary Cows’ Milk Protein A1 Beta-Casein Increases the Incidence of T1D in NOD Mice
Source: Nutrients. 2018 Sep 12;10(9):1291. doi: 10.3390/nu10091291 (PMC6163334; doi:10.3390/nu10091291)

Supplementary Figure 1

Treg-mediated suppression in the F4 generation NOD mice.
Tregs from 10-week old mice were purified using AUTOMACS and cultured with increasing ratios of Tresponder (Tresp) cells and anti-CD3 Ab. Proliferation of the Tresp cells were determined by CFSE incorporation using flow cytometry. % Suppression was then calculated as an inverse of the proliferation measured.

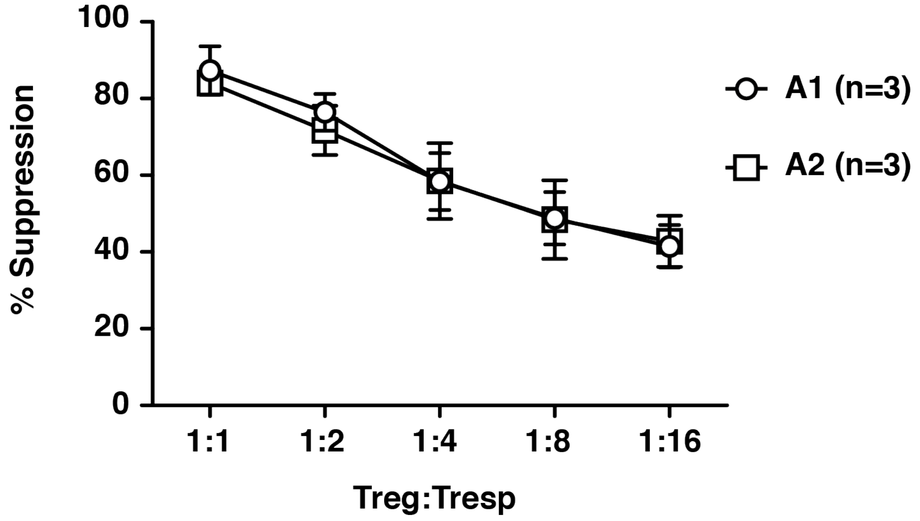

Supplement: Supplementary file 1 [file nutrients-10-01291-s001.zip › Supplementary Figure 1.pdf]
