# Supplementary figures and images for: Dietary Cows’ Milk Protein A1 Beta-Casein Increases the Incidence of T1D in NOD Mice
Source: Nutrients. 2018 Sep 12;10(9):1291. doi: 10.3390/nu10091291 (PMC6163334; doi:10.3390/nu10091291)

Alpha Diversity Measure

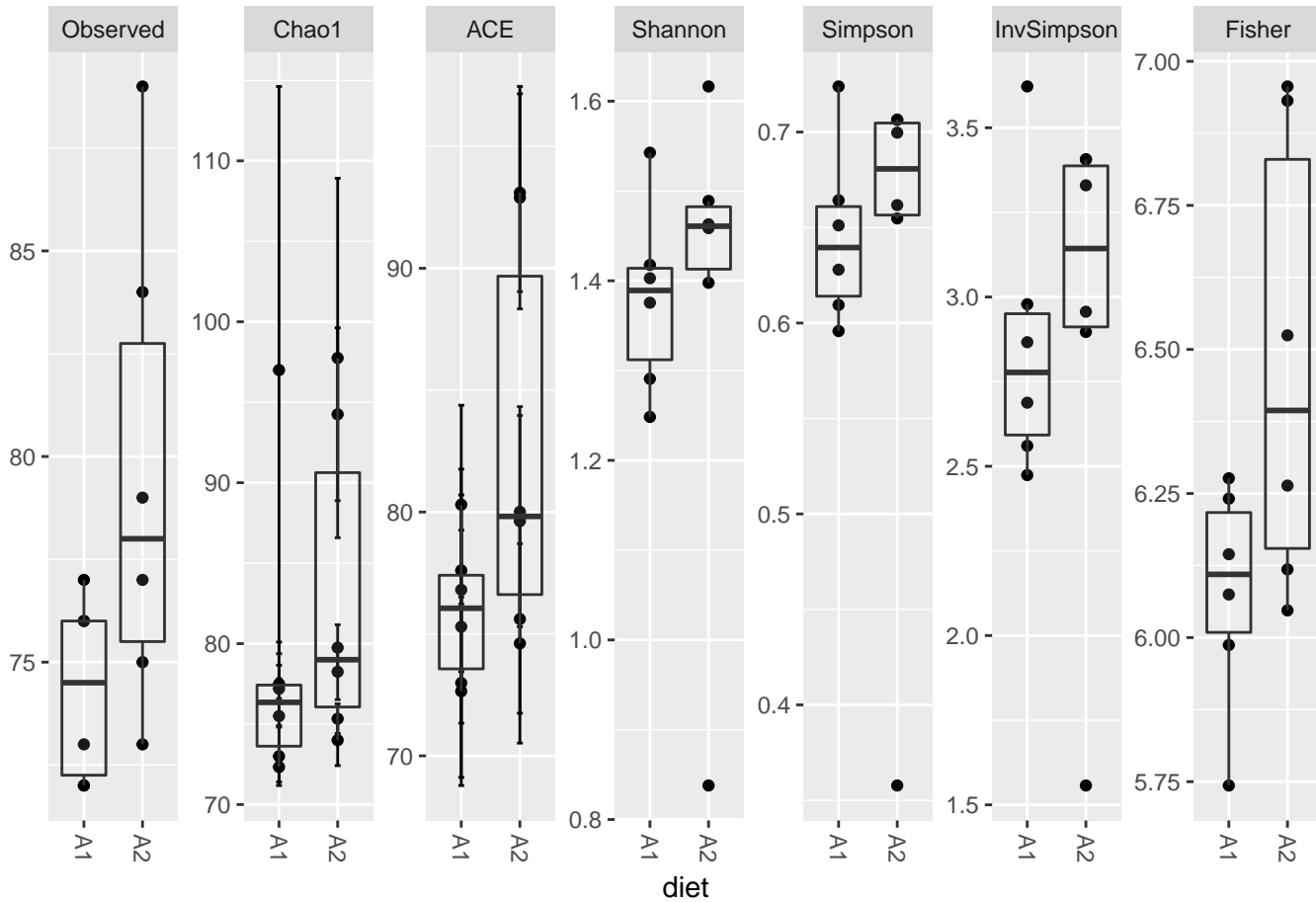

Supplement: Supplementary file 1 [file nutrients-10-01291-s001.zip › Supplementary Figure 2.pdf]
